# Atypical Functional Connectivity of the Amygdala in Childhood Autism Spectrum Disorders during Spontaneous Attention to Eye-Gaze

**DOI:** 10.1155/2012/652408

**Published:** 2012-12-27

**Authors:** Eric R. Murphy, Jennifer Foss-Feig, Lauren Kenworthy, William D. Gaillard, Chandan J. Vaidya

**Affiliations:** ^1^Department of Psychology, Georgetown University, Washington, DC 20057, USA; ^2^Children's Research Institute, Children's National Medical Center, Washington, DC 20010, USA

## Abstract

We examined functional connectivity of the amygdala in preadolescent children with Autism Spectrum Disorders (ASDs) during spontaneous attention to eye-gaze in emotional faces. Children responded to a target word (“LEFT/RIGHT”) printed on angry or fearful faces looking in a direction that was congruent, incongruent, or neutral with the target word. Despite being irrelevant to the task, gaze-direction facilitated (Congruent > Neutral) or interfered with (Incongruent > Congruent) performance in both groups. Despite similar behavioral performance, amygdala-connectivity was atypical and more widespread in children with ASD. In control children, the amygdala was more strongly connected with an emotional cognitive control region (subgenual cingulate) during interference, while during facilitation, no regions showed greater amygdala connectivity than in ASD children. In contrast, in children with ASD the amygdala was more strongly connected to salience and cognitive control regions (posterior and dorsal cingulate) during facilitation and with regions involved in gaze processing (superior temporal sulcus), cognitive control (inferior frontal gyrus), and processing of viscerally salient information (pregenual cingulate, anterior insula, and thalamus) during interference. These findings showing more widespread connectivity of the amygdala extend past findings of atypical functional anatomy of eye-gaze processing in children with ASD and challenge views of general underconnectivity in ASD.

## 1. Introduction

 Autism Spectrum Disorders (ASDs) are a class of neurodevelopmental disorders that share a trio of core symptoms: atypical social behavior, disrupted verbal and nonverbal communication, and patterns of restricted interests and repetitive behaviors. While the etiology is still unknown, there is growing consensus that ASD is a disorder of altered communication among brain regions indexed by atypical functional neural connectivity [1–3]. Functional connectivity is measured by the temporal correlation of regions visualized using functional magnetic resonance imaging (fMRI). Multiple studies have documented reduced functional connectivity in ASD, particularly between frontal and parietal cortex during social (e.g., face processing [4]; theory of mind [5]), language (e.g., sentence comprehension [6]), and executive [7] functions. Further, reduced functional connectivity in ASD subjects has also been observed during the task-free resting state [8–12]. While evidence for underconnectivity in ASD is extensive, it is not consistent as increased functional connectivity during both task and resting states has also been observed in thalamocortical [13], striatocortical [14, 15], and corticocortical [16, 17] circuits. Thus, it appears that the nature of disruption in communication in ASD likely depends upon the specific task-demands and the functional circuit it engages. 

Reduced attention towards faces and eyes, stimuli that are important for gleaning social and emotional information, is common in ASD and is thought to underlie poor social communication—a core symptom of the disorder [18]. Eye-gaze conveys information about emotional expression and mental state such as objects of interest in the environment [19, 20]. Attention towards eyes is observed in the first few months of life [21] and continues to develop as eye-gaze is used for increasingly complex social cognition, including recognition of when to begin and end social communication [22] and in referencing objects outside ones' visual field [23]. Eye-gaze remains highly salient through adulthood, as evidenced by Stroop-interference from eye-gaze when it is irrelevant to the task at hand [24–26]. This finding extends to children with ASD [25] despite observations of decreased or delayed spontaneous attention towards social stimuli [27–29]. Further, ASD subjects attend to gaze when required by the task as evidenced by intact gaze discrimination [28] and shifting of spatial attention in response to gaze [30]. However, intact behavioral performance in ASD children appears to stem from atypical engagement of underlying neural circuitry as ASD children activated regions (e.g., medial temporal lobe and dorsolateral prefrontal cortex) not activated by control children during Stroop-interference from eye-gaze [25]. Whether functional connectivity of gaze-processing regions also differs during control of attention to eye-gaze in children with ASD is not known.

 Functional brain imaging studies with typically developing individuals have identified a neural network involved in attention to social information. This network includes the amygdala, posited to encode emotional valence [31], the superior temporal sulcus (STS), posited to be involved in processing eye-gaze [32], the dorsal anterior cingulate cortex (dorsal ACC), thought to be important for assigning value to social stimuli [33], anterior insula and thalamus, posited to be important for identifying salient information, including social stimuli [34], and the posterior cingulate cortex, posited to evaluate visual information for emotional content [35]. In the ACC, the pregenual anterior cingulate cortex (pregenual ACC) is involved in the visceral response to negative stimuli, both physical and social [36], while subgenual anterior cingulate cortex (subgenual ACC) is involved in the cognitive modulation of attention towards socioemotional stimuli [37]. The orbitofrontal and ventrolateral prefrontal cortex—particularly the pars triangularis of the inferior frontal gyrus (IFG)—underlie the regulation of goal directed inhibition in response to social stimuli [38, 39]. 

We examined whether the functional connectivity of the amygdala to the distributed network of regions described above differed between 7–13 year-old children with and without ASD, during spontaneous eye-gaze processing in the context of emotional faces. Children performed a Stroop-like task in which they responded to the direction indicated by a word (LEFT or RIGHT) positioned on the nasion of faces with angry or fearful expressions [24, 26]. Across trials, faces varied in the direction of their eye-gaze such that relative to the target word, it was either congruent (leftward gaze and “LEFT” word), incongruent (leftward gaze and “RIGHT” target word), or irrelevant (neutral-central gaze and “LEFT” or “RIGHT” word). As the direction of eye-gaze is irrelevant to the task requirements (responding to the target word), it ought not to influence behavior. If it does influence behavior, however, it indicates that subjects spontaneously attended to eye-gaze. Further, contrasting the three types of trials allows examination of component processes following spontaneous attention to eye-gaze. Behaviorally, faster or more accurate responses on congruent relative to neutral trials indicate response selection that is aided by attending to eye-gaze (termed facilitation). In contrast, slower or error-prone target responses on incongruent relative to congruent trials indicate response selection in the context of resolving conflict between two response plans, one associated with the target word and the other with the interfering eye-gaze (termed interference). These predicted behavioral outcomes, interference and facilitation, provide evidence of spontaneous processing of eye-gaze with and without cognitive control, respectively. While facial expression is irrelevant to task requirements, past findings with this task indicate that the valence of facial expression modulated task performance. Interference from eye-gaze was greater for angry than fearful faces in typically developing 10–13 year-old children [24] but greater for fearful than angry faces in middle-aged healthy adults and patients with schizophrenia [26]. Thus, facial emotion potentiates the effect of eye-gaze in this task. Two negative emotions, anger and fear, were included in order to prevent subjects from habituating to a single emotion. As habituation of amygdala response to single emotions is commonly observed [40], this design attempted to minimize it. 

 We used a psychophysiological interaction analysis to assess functional connectivity of the amygdala during facilitation and interference. We restricted our examination to the network of regions discussed above, by creating an anatomical mask comprising the amygdala, superior temporal sulcus, insula, thalamus, posterior cingulate, anterior cingulate, orbitofrontal cortex, and inferior frontal gyrus. We used an anatomical mask of the left and right amygdala as seed regions, upon confirming that both groups showed activation in either the left or right amygdala during task performance relative to fixation. In order to determine whether connectivity differed by hemisphere, we directly compared left and right connectivity maps within each group. We did not have specific laterality predictions as past findings of laterality in amygdala involvement come from intentional rather than incidental encoding of social emotional information. We predicted the following regarding group differences based upon past findings. We expected children with ASD to show as much facilitation and interference as controls [25]. We predicted that amygdala connectivity would be reduced with STS [25, 41] during facilitation and would be increased with ACC and prefrontal regions during interference [25] in ASD relative to control children. These findings would parallel those from cited studies above, which show lower STS and higher prefrontal activation in ASD subjects during gaze processing and its cognitive control, respectively. Additionally, we predicted atypical connectivity of regions implicated in recognizing the salience of emotional stimuli, including the insula and thalamus based on findings suggesting abnormal amygdala activity and connectivity is related to the salience of social stimuli in ASD [42–44]. 

## 2. Materials and Methods

### 2.1. Subjects

Twelve ASD (nine males; Age: *M* = 10.42 years SD = 1.28; IQ: *M* = 116.27  SD = 15.78, measured by WISC-III) and thirteen typically developing (10 males; Age: *M* = 11.05  SD = 1.33; IQ: *M* = 115.92  SD = 13.35, estimated by WISC-III Block design and Vocabulary subtests [45]) children were paid for participation; two more ASD children participated but were excluded due to failure to withdraw stimulant medication during scanning. Children were right-handed, with Full Scale IQ above 85, and without history of seizure disorder, current antipsychotic or neuroleptic medication, and metal implants or braces; one ASD child did not complete IQ testing. There were no group differences in age (*P* = 0.26) or IQ (*P* = 0.94). In the ASD group, six children were prescribed stimulants that were withheld for at least 24 hours prior to scanning. Further, one child was on a nonstimulant ADHD medication, four children were on antidepressants and one on hypertensive medication that could not be withheld.

ASD children were diagnosed via clinical interview (by author LK) based upon DSM-IV criteria; six had diagnoses of Autistic Disorder, four of Asperger's Disorder, and two of Pervasive Developmental Disorder—Not Otherwise Specified. Diagnosis was confirmed by Autism Diagnostic Interview-Revised [ADI-R [46]] and the Autism Diagnostic Observation Schedule-Generic [ADOS-G [47]] in seven children; the remaining five children did not complete either evaluation (Table 1). Control children were screened for history of neurological and psychiatric conditions by interview, for attentional and emotional problems by Child Behavior Checklist [48], and for reading problems by Word Attack and Letter Word Identification subtests of the Woodcock-Johnson Tests of Achievement (scores > 85). All control children were screened for siblings with ASD. Further, parents of all children (except two controls and one ASD) completed the Childhood Asperger's Syndrome Test (CAST [49], a measure of the number of Asperger-like symptoms exhibited by the child. This test is a screening measure that was developed in the UK and a cut-off of 15 is recommended. As expected, scores were higher in ASD (*M* = 16.9; SD = 4.97; Range = 8–27) than in control (*M* = 4.77; SD = 2.92; Range = 1–12) children, *t*(21) = 7.5, *P* < 0.0001. For the two ASD children who scored below cut-off (score 8 and 14) and for the one ASD child who did not complete the CAST, diagnosis was confirmed with both ADI-R and ADOS-G in all three children. No control children scored above cut-off. 

### 2.2. Task Procedure

 Stimuli were created in Photoshop (Adobe Systems Inc, CA), presented in E-prime (Psychological Software Tools, Inc., PA), and viewed via a magnet-compatible projector through a mirror mounted on the head coil. Head movement was minimized with padding between the head and coil.

**Figure 1:**
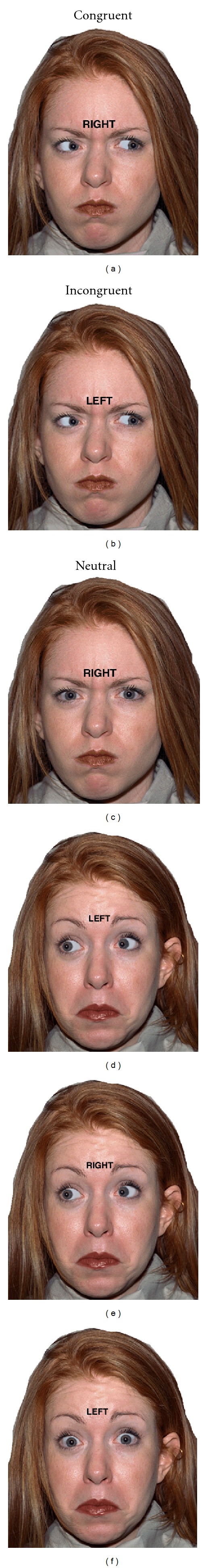
Examples of stimuli. The three columns illustrate the three conditions, congruent, incongruent, and neutral, formed by the direction indicated by the target word relative to that of eye-gaze. Top row shows faces with angry expressions and bottom row shows faces with fearful expressions.

 Subjects performed two functional runs of the Stroop-like task, with gaze as a distracter cue. Stimuli consisted of color photographs of faces of 15 individuals (7 males) showing angry or fearful emotional expression (from [50]), each with gaze directed to the left, right, or center. For two ASD children, only one run was included as the other exceeded criteria for acceptable head motion. A target word (LEFT or RIGHT) was presented in uppercase letters on the nasion of faces (see Figure 1). For the task, subjects were instructed to focus on the word and press a button as fast and accurately as possible with their right hand, index finger for “LEFT” and middle finger for “RIGHT”. On Incongruent trials gaze direction was opposite that indicated by the target word, while on Congruent trials gaze matched target word direction. For Neutral trials, gaze was directed centrally, and thus was unrelated to the target word direction. 

Each functional run lasted 325 seconds and consisted of 3 cycles, each cycle comprising three blocks of Incongruent, Congruent, and Neutral trials. The order of the three blocks was randomized in a latin-squares fashion across the three cycles. Each block consisted of 10 trials and lasted for 25 seconds; in order to minimize the predictability of the type of upcoming trials, two Neutral trials were interspersed randomly in the Incongruent and Congruent blocks. Stimuli within blocks consisted entirely of one emotion (Anger or Fear). The two Neutral trials within Incongruent and Congruent blocks maintained the emotional content of the other stimuli in the block. Each trial consisted of presentation of the visual display for 1000 ms, followed by a 1500 ms lag. Blocks were separated by 5 fixation trials lasting 12.5 seconds. Two runs of the task were performed, each containing fear and anger blocks resulting in three blocks of each combination of emotion and congruency across the two runs.

### 2.3. Imaging Procedure

 A high-resolution sagittal T1-weighted scan was acquired on a Siemens Trio 3.0T MRI scanner using a 3D MPRAGE sequence with a scan time of 6:51 min and the following parameters: TR = 1600 ms, TE = 4.4 ms, 256 × 256 mm FOV, 160 mm slab with 1 mm thick slices, 256 × 256 × 160 matrix (effective resolution is 1.0 mm^3^), 1 excitation, and a 15 degree flip angle. Functional images were acquired using a *T*_2_*-sensitive gradient echo pulse sequence with the following parameters: TR = 2500 ms, TE = 30 ms, 256 × 256 mm FOV, 64 × 64 acquisition matrix, and a 90 degree flip angle. Forty-two 4 mm thick slices were acquired descending in the transverse plane for 132 time points (the first 2 TRs were included for signal stabilization and discarded from analysis).

### 2.4. Preprocessing

Data were analyzed using SPM8 and its custom toolboxes (Wellcome Department of Cognitive Neurology, London, UK). Images were realigned and motion-corrected using the INRealign toolbox [51]. All included subjects had less than 4 mm of head movement in *x*, *y*, or *z* directions. However, even small amounts of motion (e.g., 1 mm) can cause errors in image reconstruction and spin history that are particularly problematic for functional connectivity analysis. Therefore, custom methods used in other pediatric studies [52] were used to minimize motion artifact using the ArtRepair toolbox [53]. Volumes with excessive scan-to-scan motion and large global signal change (defined as 0.5 mm/TR and 1.5% SD from grand mean signal) were identified and repaired based on a linear interpolation from nearest subthreshold volumes. However, this method is likely to produce artifactual activation in the event that a large number of volumes (e.g., 30%) require repair. Thus, for those subjects (2 ASD, 3 Control), motion artifact components were identified with Independent Component Analysis [54] using the Melodic toolbox in FSL [55]; these components were included as regressors of no interest during model estimation. Motion corrected data were then normalized into the MNI EPI template and resampled to 4 × 4 × 4 mm^3^ voxels. Normalization of pediatric samples to adult stereotactic space has been validated in children as young as 6 years [56]. Normalized image volumes were spatially smoothed using a 10 mm full width at half-maximum Gaussian kernel and temporally filtered (high-pass filter: SPM default 128 s). fMRI responses were modeled by canonical hemodynamic response function with a boxcar function lasting for the duration of each block. To maximize statistical power, blocks were collapsed across emotion, such that task blocks consisted only of Neutral, Congruent, and Incongruent conditions.

### 2.5. Functional Connectivity

Functional connectivity was analyzed using the Psychophysiological Interaction (PPI) toolbox in SPM8. Amygdala ROIs were created based on the AAL [57] brain atlas with the MarsBaR toolbox for SPM [58]. First and second runs were analyzed separately to avoid introducing potential motion or signal artifacts into the time series analysis. For each run, the first eigenvariate time series of activity was then extracted from normalized nonsmoothed data (to avoid signal contamination from nonamygdala brain regions) for all voxels within the seed ROI. For each subject, a design matrix was created in which one regressor represented the deconvolved eigenvariate of the amygdala seed region, a second regressor represented the task contrast of interest (facilitation (Congruent > Neutral) or interference (Incongruent > Congruent)), and a third regressor represented the cross-product of these two regressors. This interaction term, the PPI regressor, was then used as a template to interrogate similar task-related activity patterns—functional connectivity—across the brain.

Results from individual subject PPI analyses were used to conduct second-level analysis restricted to a bilateral anatomical mask encompassing the cingulate gyrus, STS, OFG, IFG, insula, and thalamus created using the WFU pickatlas [59]. For facilitation and interference separately, connectivity maps for the right and left amygdala were compared within each group with a paired *t*-test to identify laterality differences. Further, for the right and left amygdala maps, separately, groups were compared with two-sample *t*-tests. For both within and between group comparisons, a threshold of *P* < 0.01, *k* = 37 was used, which is a significance level of *P* < 0.05 corrected for multiple comparisons based on Monte Carlo simulation of random noise distribution (using 3dClustSim module of AFNI [60].

## 3. Results

### 3.1. Behavior

For each subject, correct left and right key presses were combined to compute percent error and mean response latency for accurate Incongruent, Congruent, and Neutral trials (Figure 1). Group X Trial-type analyses of variance (ANOVAs) were computed to assess group differences in facilitation (Congruent versus Neutral) and interference (Incongruent versus Congruent), separately for reaction time and percent accuracy. Average total omission rate was 0.85% for controls and 1.46% for ASD, with no group differences seen in omitted responses (*P* = 0.38), and no subject omitting more than two responses in any task block (Table 2).

**Facilitation (Congruent versus Neutral)**

A main effect of Trial-type revealed significant facilitation, decreased response time, *F*(1, 23) = 6.53, *P* = 0.02 and fewer errors, *F*(1, 23) = 8.33, *P* = 0.003, for Congruent than Neutral trials. Group differences did not reach significance for either response time (*P* = 0.15) or accuracy (*P* = 0.65). Further, Group X Trial-type interaction was also not significant for response time (*P* = 0.45) or accuracy (*P* = 0.79). Thus, attention to eye-gaze facilitated responses similarly for ASD and control children.

**Interference (Incongruent versus Congruent)**

A main effect of Trial-type revealed significant interference, increased response time, *F*(1, 23) = 23.49, *P* < 0.001 and increased errors, *F*(1, 23) = 9.23, *P* = 0.006, for Incongruent than Congruent trials. Group differences did not reach significance for accuracy (*P* = 0.77), though response time was slightly slower for ASD subjects (*P* = 0.04). However, Group X Trial-type interaction was not significant for response time (*P* = 0.31) or accuracy (*P* = 0.61). Thus, attention to eye-gaze interfered with responses to target words similarly in ASD and control children.

### 3.2. Functional Connectivity

#### 3.2.1. Laterality Differences

 Results are listed in Table 3 by anatomy, Brodmann Area, MNI coordinates, and *Z* value.

**Facilitation (Congruent versus Neutral)**

**Figure 2:**
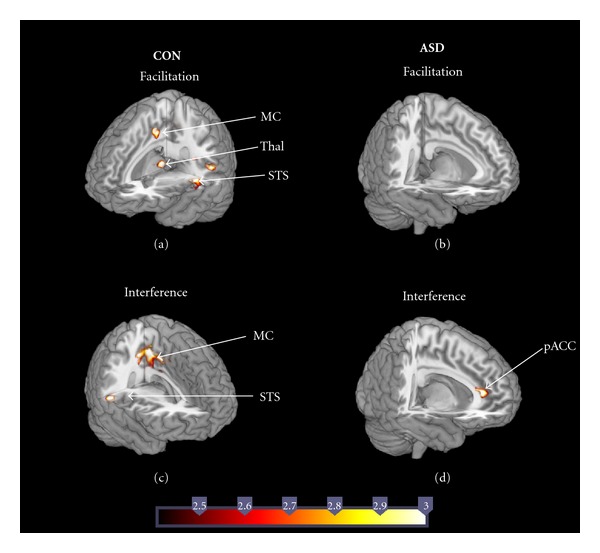
Regions showing differences between left and right amygdala connectivity in control (CON) and ASD children during facilitation (Congruent > Neutral) and interference (Incongruent > Neutral). In control children, greater right than left amygdala was observed during facilitation (a) but greater left than right amygdala connectivity was observed during interference (c). ASD children showed greater right than left amygdala connectivity during both facilitation (b) and interference (d).

Relative to the right amygdala, control children showed increased connectivity of the left amygdala with left STS (BA 22, 42), bilateral thalamus, and the middle cingulate gyrus, often called motor cingulate (BA 24, 31) (Figure 2(a)). No regions showed greater right relative to left amygdala connectivity in controls. In children with ASD, no regions showed significantly different connectivity between right and left amygdala (Figure 2(b)).

**Interference (Incongruent versus Congruent)**

Control children showed increased connectivity of the right amygdala with motor cingulate (BA 24, 31) and right STS (BA 41, 42) relative to that of the left amygdala (Figure 2(c)). No regions showed greater left than right amygdala connectivity in controls. In children with ASD, the right amygdala showed increased connectivity with the pregenual ACC (BA 24, 32) (Figure 2(d)). No regions showed greater left than right amygdala connectivity in ASD children.

In sum, connectivity patterns of the left and right amygdala differed between ASD and control children such that control children showed higher connectivity for the left amygdala during facilitation and for the right amygdala during interference, whereas children with ASD showed no difference in left and right amygdala connectivity during facilitation, while showing higher connectivity of the right amygdala during interference.

#### 3.2.2. Group Differences

 Results of the group comparisons are listed in Table 4, by anatomy, Brodmann Area, MNI coordinates, and *Z* value.

**Facilitation (Congruent versus Neutral)**

**Figure 3:**
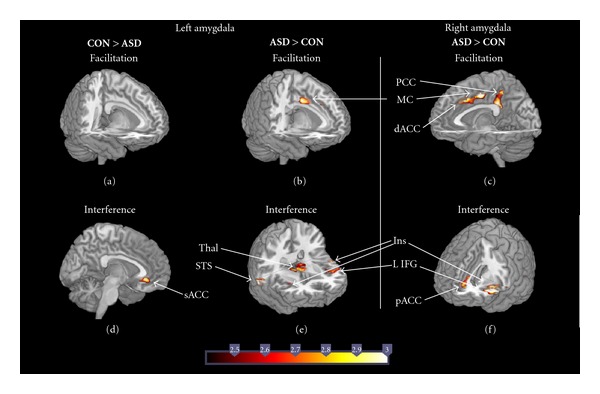
Regions showing differences between ASD and control children for left and right amygdala connectivity during facilitation (Congruent > Neutral) and interference (Incongruent > Neutral). In control children, only left amygdala showed increased connectivity relative to ASD in interference (d), while ASD showed increased connectivity in both amygdalae relative to controls during both facilitation (b) and (c), and interference (e) and (f).

Amygdala connectivity was not higher in control than ASD children in any region during facilitation (Figure 3(a)). Relative to control children, ASD children showed increased right amygdala connectivity with several regions along the cingulate cortex, with two peaks in posterior cingulate (one anterior (BA 23) and another slightly posterior and superior to it (BA 5, 24)), one in motor cingulate (BA 24) and one in dorsal ACC (BA 24, 32) (Figure 3(c)). Further, children with ASD also showed increased left amygdala connectivity with motor cingulate (BA 24) relative to control children. (Figure 3(b)).

**Interference (Incongruent versus Congruent)**

Relative to children with ASD, control children showed increased left amygdala connectivity with subgenual ACC (BA 32) (Figure 3(d)); right amygdala connectivity was not higher in control than ASD children in any region. Relative to control children, ASD children showed increased right amygdala connectivity with pregenual ACC (BA 24, 32), left insula (BA 13), and left IFG (Figure 3(f)). A subset of these regions, namely left insula, and left IFG (BA 44, 45), along with right insula (BA 13), bilateral STS (BA 22), and bilateral thalamus also showed increased connectivity with left amygdala in ASD relative to control children (Figure 3(e)).

In sum, connectivity of the amygdala during facilitation and interference associated with eye-gaze was more widespread in ASD children relative to controls, involving regions of the cingulate gyrus, insula, and ventral-lateral prefrontal cortex. 

## 4. Discussion

Functional connectivity of the amygdala during spontaneous attention to eye-gaze in emotional faces with and without cognitive control, differed between ASD and control children, despite similar behavioral performance. While control children's amygdala was more strongly connected to socioemotional cognitive control (subgenual ACC) regions during interference, ASD children's amygdala was more strongly connected to multiple regions implicated in processing of salient information and cognitive control during both facilitation and interference. These regions included dorsal ACC, motor cingulate, and posterior cingulate gyrus during facilitation and pregenual ACC, insular and ventrolateral prefrontal cortex, thalamus, and STS during interference. Thus, amygdala connectivity was both atypical and more widespread in children with ASD. Further, direct comparison of left and right amygdala connectivity showed a different pattern of lateralization between the groups: control children had stronger connectivity for the left amygdala during facilitation but right amygdala during interference, whereas ASD children showed no lateralization of amygdala connectivity during facilitation, but did show stronger right amygdala connectivity during interference. Together, these findings shed light upon integrated processing of the amygdala with fronto-temporal regions during spontaneous attention to eye-gaze in faces in typical development and how it differs in ASD.

Our behavioral findings indicate that children with ASD attended spontaneously to eye-gaze in faces to the same extent as control children. The task, responding to the direction indicated by words (LEFT or RIGHT), does not require attention to any aspects of the contextual facial stimuli, except words printed on the nasion. However, if the direction of eye-gaze in the faces influences children's task performance, it provides behavioral evidence for incidental or spontaneous encoding of eye-gaze. Indeed, response speed and accuracy were influenced by eye-gaze such that they were facilitated on congruent trials and impeded on incongruent trials. Most importantly, the magnitude of these effects did not differ between groups, indicating that children with ASD paid as much attention to eye-gaze as control children. Failure to control for the amount of attention to faces has been proposed as an important reason for disparate findings of hypo- or hyperactivation of face and emotion processing regions in ASD subjects in past work [42]. Our paradigm addresses this concern by providing a behavioral proxy, by measuring the incidental influence of gaze-direction on an ongoing word classification task. As a result, amygdala connectivity in the present study can be interpreted in the context of processing eye-gaze. The extent to which emotional information influenced amygdala engagement, however, cannot be discerned in the present study. In past studies using this task, we have found that interference is sensitive to emotional valence such that it was higher for angry than fearful faces in preadolescent children than adults [24] and higher for fearful than angry faces in middle-aged schizophrenic patients and controls [26]. We did not have enough trials in the present task, which was shortened for fMRI relative to a behavioral study, to reliably compare average performance for fearful and angry faces. Thus, whether observed connectivity findings reflect the influence of emotional expression cannot be confirmed. 

The observed hemispheric differences in amygdala connectivity extend current knowledge about the roles of the left and right amygdala. Numerous studies have shown lateralized amygdala activation during a variety of tasks involving exposure to emotional stimuli (for review see [61]) leading to different putative functional roles for the right and left amygdala. Specifically, the right amygdala is thought to underlie a fast and shallow dynamic stimulus detection mechanism, whereas left amygdala is thought to underlie sustained evaluation of stimuli [62]. This hypothesis of lateralized amygdala function has been directly supported by findings of differential amygdala habituation to repeated emotional faces [63], as well as by a meta-analysis that evaluated amygdala lateralization effects by task [64], showing that tasks involving rapid emotional processing, such as masked stimuli, were more likely to activate the right amygdala, while tasks involving effortful or sustained emotional processing, such as reading emotional words, were more likely to activate the left amygdala. 

In the context of the above view, observed hemispheric differences within each group suggest that children with ASD failed to habituate during facilitative gaze-processing and to engage cognitive control processes during interfering gaze-processing. During facilitative eye-gaze processing, control children had higher left (relative to right) amygdala connectivity with MC, left superior temporal sulcus, and bilateral thalamus. This result coupled with no regions showing greater right than left amygdala connectivity suggests that right amygdala connectivity was attenuated due to habituation to face stimuli, while the left amygdala maintained functional connectivity to regions relevant to ongoing task demands. Specifically, the STS is involved in encoding eye-gaze [50] and the MC is involved in motor responses. Further, during processing interfering eye-gaze, control children had higher right (relative to left) amygdala connectivity with the motor cingulate and right STS. This pattern suggests a lack of habituation to the face stimuli, a task-appropriate response for a condition in which conflict has to be resolved between responses triggered by the target word and task-irrelevant eye-gaze cues. In contrast to control children, ASD children did not show laterality differences during processing of facilitative eye-gaze. This result, showing equal levels of connectivity between left and right amygdala, indicates a lack of right-amygdala habituation when eye-gaze is beneficial. Further, during processing of interfering eye-gaze, ASD children had higher right (relative to left) amygdala connectivity with pregenual ACC, a region involved in processing salient information. While this result suggests a lack of habituation of right amygdala, a task-appropriate response for a condition requiring cognitive control, it also shows engagement of a task-unnecessary region—rather than maintaining greater connectivity with cognitive control regions such as motor cingulate, children with ASD maintained greater connectivity with regions encoding salience. This finding of increased salience network recruitment when attending to emotional faces is consistent with findings in adolescents [43] and adults [42] with ASD, both showing increased activation of the right amygdala, as well as salience processing regions such as the insula, to briefly presented emotional faces relative to controls despite equal attention to the faces. 

Findings from directly comparing groups during facilitation suggest different routes to processing of eye-gaze in ASD and control children. While gaze perception was not impaired in children with ASD in the present study, their gaze processing involved greater connectivity of the amygdala with dorsal anterior, and posterior aspects of the cingulate gyrus (motor cingulate, dorsal ACC, and posterior cingulate). The posterior cingulate has been implicated in the processing of emotionally salient stimuli [65], particularly in evaluation of visual information for emotional content [35], while dorsal ACC involvement has been associated with the monitoring of conflict from both emotional and nonemotional [66] stimuli. As congruent trials did not involve conflict, the finding of increased dorsal ACC-amygdala connectivity in ASD subjects might appear anomalous. However, individuals with alexithymia—who also have difficulty with understanding and differentiating emotions in social interactions—engage dorsal ACC while viewing highly arousing images in a task that did not involve resolving conflict; further, this engagement was positively correlated with both amygdala activation and ratings of arousal [67]. Heinzel et al. [67] speculated that this may represent an effort to downregulate the arousal of the emotional content by recruiting cognitive resources beyond those typically involved in emotional control. In ASD, increased skin conductance when viewing direct or indirect eye-gaze [68], similarly suggests increased autonomic arousal when viewing these stimuli. Perhaps increased connectivity of the amygdala with dorsal ACC in children with ASD reflects regulatory processes invoked during facilitative eye-gaze processing. It would be worthwhile to test this experimentally in the future. 

Findings from directly comparing groups during interference, a condition requiring cognitive control of eye-gaze processing, revealed a widespread functional connectivity network associated with the amygdala in children with ASD. Control children showed greater amygdala connectivity with the subgenual ACC, a region known to be engaged during tasks involving cognitive control in the emotional domain such as conflict resolution [69] and monitoring and regulating emotional and visceral states [70]. In contrast, children with ASD exhibited amygdala connectivity with a network including pregenual ACC, bilateral insula, thalamus, and STS, and left IFG. Pregenual ACC has been linked with visceral nocioceptive activity, particularly an increase in unpleasantness during noxious stimulation [36]. Specifically, functional connectivity between pregenual ACC, thalamus, and anterior insula has been posited as an emotional salience monitoring system whose purpose is to quickly attune an individual to salient features within incoming sensory stimuli [34] and to integrate interoceptive information with emotional salience to assess bodily states [71]. Therefore, stronger connectivity of the amygdala with this network rather than subgenual ACC in ASD may reflect maintenance of that aversive state. Atypical engagement of pregenual ACC and the frontoinsular cortex may be associated with the abnormal development of von Economo neurons which are known to be increased in number and ratio relative to pyramidal neurons in the frontoinsular cortex in ASD [72]. As IFG activity has been related to inhibitory control over motor responses to emotional cues [39], increased connectivity in ASD relative to controls suggests that the lack of emotional modulation by the subgenual ACC effectively shifted the cognitive burden from emotional regulation to motor regulation in an emotional context.

One limitation of the current study is that we have no behavioral support for positing hyperactivity of an emotional salience network as we did not measure emotional reactivity. Thus, it is important to garner behavioral support for our speculation in future work. Second, while our sample of 12 ASD subjects is small, this size is not unusual in past ASD imaging work (72: *N* = 11; 73: *N* = 10; 74: *N* = 8; 75: *N* = 8), though replication of these findings in a larger sample is necessary. Third, the extent to which the observed group differences relate to volumetric differences in the amygdala is unknown. Past studies have shown bilateral volumetric differences between ASD and control subjects at various ages [73, 74]. Future work should examine the relationship between amygdala volume and abnormal connectivity.

## 5. Conclusions

The current findings suggest that children with ASD display disordered functional connectivity in networks that underlie both the ascription of salience to social stimuli and those that modulate attention to those stimuli. While decreased amygdala connectivity relative to controls was seen in regions expected to mediate interfering gaze, greater amygdala connectivity was seen in several regions, including areas implicated in salience processing. While these results do not suggest a potential cause of this disordered functional connectivity, they do provide evidence counter to the hypothesis that ASD is characterized by global underconnectivity. The current findings are in line with previous ones that individuals with ASD show increased activity in salience detection regions such as the right amygdala and insula [42, 43], and increased autonomic response when looking at emotional faces [68], as well as impaired connectivity of regions necessary for the cognitive control of emotional attention [9]. We therefore propose that deficits in social interaction in ASD may be the result of inefficient processing of facilitative social cues coupled with an increased ascription of salience to stimuli that may be perceived as socially incongruent. 

## Figures and Tables

**Table 1:** Demographic information for ASD children.

	Gender	DX	FSIQ	VIQ	PIQ	ADI Soc.	ADI Comm	ADI Rep. Beh.	ADOS Comm + Soc.	CAST
Subject 1	M	ASP	124	146	95	20	14	9	13	16
Subject 2	M	ASD	88	102	77	25	24	10	12	27
Subject 3	F	ASD	133	118	138	22	24	8	8	8
Subject 4	M	ASP	121	108	130	23	15	5	14	21
Subject 5	M	ASP	85	96	78	24	21	10	15	14
Subject 6	M	ASD	126	126	120	—	—	—	—	16
Subject 7	M	ASD	119	118	119	27	22	12	12	20
Subject 8	F	ASP	132	140	129	—	—	—	—	15
Subject 9	M	PDD-NOS	118	117	116	19	17	7	5	—
Subject 10	M	ASD	117	103	127	—	—	—	—	16
Subject 11	F	PDD-NOS	116	119	108	—	—	—	—	16
Subject 12	M	ASD	—	—	—	—	—	—	—	18

**Table 2:** Percentage of correct and omitted responses for each trial type in ASD and control children.

	Congruent	Incongruent	Neutral
% Correct responses			
Control	94.07	92.47	91.35
ASD	95.83	90.72	92.24
% Omitted responses			
Control	0.13	0.19	0.11
ASD	0.25	0.18	0.30

**Table 3:** Regions showing differences between right and left amygdala connectivity in control (CON) and ASD children during facilitation (Congruent > Neutral) and interference (Incongruent > Congruent) from eye-gaze (*P* < 0.05 Monte Carlo corrected).

Group	Condition	Contrast	Region	BA	*X*	*Y*	*Z*	*Z* value
CON	Facilitation	Left > Right	Motor cingulate	31, 24	3	−13	49	4.03
			L STS	22	−42	−22	1	3.44
			L Thalamus		−3	−22	7	3.24
			R Thalamus		3	−25	4	3.41
			L STS	42	−51	−31	16	2.98
	Interference	Right > Left	None					
		Left > Right	None					
		Right > Left	Motor cingulate	31, 24	−15	−43	52	3.4
			R STS	41, 42	60	−31	10	3.22

ASD	Facilitation	Left > Right	None					
		Right > Left	None					
	Interference	Left > Right	None					
		Right > Left	Pregenual ACC	24, 32	−3	35	10	3.25

**Table 4:** Regions showing group differences in left and right amygdala connectivity between control (CON) and ASD children during facilitation (Congruent > Neutral) and interference (Incongruent > Congruent) from eye-gaze (*P* < 0.05 Monte Carlo corrected).

Condition	Hemisphere	Contrast	Region	BA	*X*	*Y*	*Z*	*Z* value
Facilitation	Left Amyg	CON > ASD	None					
		ASD > CON	MC	24	−9	−7	37	3.08
	Right Amyg	CON > ASD	None					
		ASD > CON	Posterior cingulate	23	−3	−34	28	3.22
			Posterior cingulate	5, 24	−15	−40	52	3.59
			Dorsal ACC	24, 32	−3	20	37	3.13
			Motor cingulate	24	0	−7	46	3.54

Interference	Left Amyg	CON > ASD	Subgenual ACC	32	6	35	−8	3.09
		ASD > CON	R Insula	13	33	14	10	2.96
			L Insula	13	−42	8	−2	2.88
			L STS	22	−60	−1	4	3.10
			R STS	22	69	−25	1	3.45
			L IFG	44, 45	−57	17	7	2.68
			L Thalamus		−6	−10	7	2.87
			R Thalamus		3	−19	10	3.09
	Right Amyg	CON > ASD	None					
		ASD > CON	Pregenual ACC	24, 32	−6	41	7	2.90
			L IFG	45, 47	−48	26	4	3.43
			L Insula	13	−45	5	1	2.77
